# Preparation of Heterogeneous Fenton Catalysts Cu-Doped MnO_2_ for Enhanced Degradation of Dyes in Wastewater

**DOI:** 10.3390/nano14100833

**Published:** 2024-05-09

**Authors:** Xiao Liu, Lu Wang, Jiran Li, Rong Li, Runze He, Wanglong Gao, Neng Yu

**Affiliations:** 1 School of Materials Science & Engineering, North Minzu University, Yinchuan 750021, China; luwang111222333@163.com (L.W.); 18314293192@163.com (J.L.); lr157821@163.com (R.L.); 15591779243@163.com (R.H.); chenyielegant@163.com (W.G.); 2 Key Laboratory of Polymer Materials and Manufacturing Technology, North Minzu University, Yinchuan 750021, China; 3 International Scientific and Technological Cooperation Base of Industrial Waste Recycling and Advanced Materials, Yinchuan 750021, China; 4 Huadian Electric Power Research Institute Company, Hangzhou 310012, China; neng-yu@chder.com

**Keywords:** Cu-doped MnO_2_, nanocatalyst, Congo red degradation, heterogeneous Fenton reaction

## Abstract

Herein, a series of heterogeneous Fenton catalysts, Cu doped MnO_2_ (CDM), with different Cu/Mn molar ratios were prepared via a hydrothermal reaction. Meanwhile, detailed characterizations were used to study the structures of CDM, and it is amazing that the morphology of CDM changed from nanowires to nanoflowers with an increasing amount of Cu doped. Apart from this, both the specific surface area and oxygen vacancy increased obviously with the increasing Cu/Mn molar ratio. Then, the degradation of different dyes was utilized to evaluate the catalytic activity of different CDM with H_2_O_2_ used as the oxidizing agent, and the 50%-CDM with the highest content of Cu doped displayed the best catalytic activity. Herein, the degradation efficiency (D%) of Congo red (CR) solution with low concentration (60 mg/L) reached 100% in 3 min, while the D% of CR solution with a high concentration (300 mg/L) reached 99.4% after 5 min with a higher dosage of H_2_O_2_. Additionally, the 50%-CDM also displayed excellent reusability, for which the D% values were still higher than 90% after the 14th cycles. Based on the structure characteristics and mechanism analysis, the excellent catalytic capacity of 50%-CDM was due to the combined influence of large specific surface area and abundant oxygen vacancy. Thus, a promising heterogeneous Fenton catalyst was developed in this study, which proved the treatment efficiency of actual dye wastewater.

## 1. Introduction

With the continuous development of industrialization, a large amount of dye wastewater containing refractory organics is produced. It has been shown that more than 15% of dyes are discharged into the natural environment directly, and these have brought serious environmental problems and health risks [1,2]. In particular, some high-use dyes, such as CR, methyl blue (MBE), rhodamine B (Rh B), and methylene blue (MB), have more stable, nonbiodegradable, aromatic molecular structures that pose a serious threat to the environment [3]. Thus, the development of efficient and sustainable technology for dye wastewater treatment is very urgent [4,5]. Among the various technologies utilized until now, the heterogeneous Fenton reaction is regarded as an efficient and promising approach. The heterogeneous Fenton reaction is a kind of advanced oxidation process (AOP) [6], which can generate •OH and •O_2_^−^ from H_2_O_2_ under the catalysis of nanocatalysts and can cause the degradation of organic pollutants further [7,8,9]. Compared with the traditional homogeneous Fenton reaction, the heterogeneous Fenton reaction displays smaller quantities of sludge, wider pH applicability, higher catalytic performance, and so on, for which it has attracted much attention [10,11]. 

In the development of a heterogeneous Fenton reaction, the key problem is to develop high-activity nanocatalysts. MnO_2_, as a metal oxide, has been used widely as a nanocatalyst [12] due to its unique properties such as multiple valences of manganese, low toxicity, large surface area, primary adaptability, strong adsorption, and catalytic performance [13,14]. For example, Li et al. prepared highly porous α-MnO_2_ nanorods by selective acid etching from Mn-containing spinel, and 90.9% of 4-chlorophenol can be degraded within 12 min by catalytic ozonation in a wide range of pH of 4.5–10.5 [15]. Wang et al. synthesized three-dimensional hierarchical (3D) MnO_2_ via the calcination of hydrothermal products and utilized MnO_2_ to degrade acid orange 7, and the 3D MnO_2_ delivered great catalytic performance due to its high specific surface area [16]. Lu et al. prepared MnO_2_ with different morphologies, including nanorod, nanoflower, nanowire, and nanourchin, and the results proved that the nanoflower MnO_2_ displayed the highest catalytic activity. Therefore, this is more due to better oxidative and easier oxygen migration rather than just a high specific surface area [17]. He et al. evaluated the catalytic activity of MnO_2_ with different crystal phases and the following results were obtained: α-MnO_2_ > β-MnO_2_ > γ-MnO_2_ [18]. However, some different results about the structure–activity relationship of MnO_2_ have been reported in other studies [1,19,20], so He et al. indicated that it is still hard to confirm the remarkable factors influencing the catalytic activity of MnO_2_ due to the different preparation methods of catalysts [18]. 

However, the results obtained in previous studies indicated that the crystalline phase and morphologies played important roles in the catalytic activity of MnO_2_. As a result, MnO_2_ nanocatalysts with higher activity could be produced by novel preparation methods, and intercalation chemistry is a highly effective method to improve the catalytic performance of MnO_2_ [21]. Wang et al. compared the activity of a Co(II)-intercalated δ-MnO_2_ (Co-δ-MnO_2_) catalyst and original δ-MnO_2_ catalyst; the degradation efficiency of δ-MnO_2_/PMS was 60.1%, while that of Co-δ-MnO_2_/PMS reached 100% in the same reaction conditions [22]. Yu et al. synthesized Cu-doped δ-MnO_2_@diatomite to degrade the MB, and the catalyst had excellent oxidation ability to dissociate H_2_O_2_ to a hydroxyl radical [23]. Yang et al. prepared metal-doped amorphous MnO_2_ (M-AMO, M = Fe, Co, Ni, and Cu) for organic oxidation with PMS, and the Cu-AMO displayed the highest activity compared to the other metal-doped MnO_2_, for which the rate constant reached 3.5 times as high as that of pure MnO_2_ [24]. According to the above reports, the high catalytic activity could be attributed to two respects: one is that the metal doping had a significant impact on the surface electron and charge transfer by the creation of more oxygen vacancies, the other is that the change in catalyst surface morphology promotes the interaction between the catalyst, the oxidizing species, and the organic pollutant. Although many studies about metal-doped MnO_2_ have been investigated, deep research about the effect of Cu doping on the structure and catalytic activity of MnO_2_ has been reported rarely, and the mechanism of its structure–activity relationship also needs to be analyzed further.

In this study, various CDM with different contents of Cu were prepared by a simple hydrothermal reaction, and a series of characterizations were utilized to investigate the structures of CDM. To evaluate the catalytic activity of CDM, a CR degradation reaction was taken out by a heterogeneous Fenton reaction with activating H_2_O_2_. Finally, the catalytic mechanisms of CDM were discussed according to the experimental results of a free radical scavenging test and EPR measurement. On account of the large specific surface area and abundant oxygen vacancy, the Cu-doped MnO_2_ displayed an outstanding degradation capacity of CR. Thus, a heterogeneous catalyst with high catalytic activity and good operation performance was obtained herein, which proved the treatment efficiency of a catalytic oxidation system for the actual dye wastewater.

## 2. Materials and Methods

### 2.1. Materials

Potassium permanganate (KMnO_4_) and acetic acid were purchased from Shanghai Wokai Biotechnology Co. LTD (Shanghai, China), copper sulfate (CuSO_4_), ammonia, CR, methyl blue (MBE), rhodamine B (Rh B), and methylene blue (MB) were obtained from Guangzhou Chemical Reagent Factory (Guangzhou, China). All other chemicals used in the experiments were purchased from China National Pharmaceutical Group Corporation (Beijing, China), and all of the reagents were chemical grade.

### 2.2. Synthesis Procedures

A simple hydrothermal reaction was utilized to prepare a CDM nanocatalyst according to our previous study [12]. Firstly, 0.6 g of KMnO_4_ was dissolved into 30 mL 0.4 M acetic acid with stirring at room temperature and a certain amount of CuSO_4_·5H_2_O was dissolved in 10 mL of distilled water. Then, the two solutions were mixed thoroughly and transferred to a 50 mL Teflon-lined autoclave with 40 µL ammonia added. Finally, the CDM nanocatalyst with different Cu/Mn molar ratios was obtained after a hydrothermal reaction at 140 °C for 12 h. Additionally, a pure MnO_2_ nanocatalyst was also prepared according to the above method without CuSO_4_·5H_2_O. Thus, a series of nanocatalysts with different compositions were prepared, as shown in Table 1.

### 2.3. Characterization

The morphologies and elemental mapping of series catalysts were observed by scanning electron microscopy (SEM, Zeiss, sigma500, Jena, Germany) with an Oxford Ultim Max Large Area SDD EDS detector. The size of catalysts was measured by a laser particle size analyzer (Malvern, MS-2000, Malvern, UK). The crystallographic structures of catalysts were characterized by a high-resolution transmission electron microscope (HRTEM, JEM-2100, Tokyo, Japan) and X-ray diffraction (XRD, Rigaku Corporation, XRD-6000, Tokyo, Japan). The specific surface area and pore size distribution of samples were analyzed by Brunauer–Emmett–Teller (BET) and Barrett–Joyner–Halenda (BJH) methods, respectively. A Fourier-transform infrared spectrophotometer (FT-IR, American Nicolet Corp. Model 170-SX, Green Bay, WI, USA) was used to investigate the chemical structures of the catalyst. X-ray photoelectron spectroscopy (XPS, Thermo Fisher Scientific, ESCALAB250Xi, Waltham, MA, USA) was utilized to study the surface chemical composition of catalysts. To study the catalytic mechanism of CDM, an electron paramagnetic resonance spectrometer (Bruker ELEXSYS E500, Karlsruhe, Germany) was used to obtain the EPR signals. The leaching of Cu and Mn in wastewater was detected by an inductively coupled plasma spectrometer (Agilent 5800 ICP-OES, Santa Clara, CA, USA).

### 2.4. Heterogeneous Fenton Degradation of Dyes

To study the catalytic ability of CDM that were prepared, dye degradation experiments were taken out, and different dyes were selected, including MBE, CR, Rh B, and MB. In a typical experiment, 0.05 g of CDM and 2 mL of 30% H_2_O_2_ were added into 50 mL of dye solution (60 mg/L), and the reaction was taken out in a shaker (150 rpm/min) for a certain time. When the degradation reaction finished, the remaining dye concentration was calculated by measuring the absorbance of dye solution using a UV-vis spectrophotometer at a certain wavelength (668 nm for MBE, 498 nm for CR, 552 nm for Rh B, and 664 nm for MB). And D% was calculated using the following equations [12]:

(1) $$D\%=\frac{C_{o}-C_{t}}{C_{t}}\times 100$$

Herein, *C_o_* and *C_t_* (mg/L) are the initial and instantaneous concentrations of dye wastewater, respectively. All of the degradation experiments were tested in triplicate to reduce experimental error.

To investigate the optimal conditions for degradation reactions, the influences of the initial CR concentration, H_2_O_2_ dosage, pH of the solution, and temperature on the degradation of CR were investigated. Additionally, the reusability of catalysts was also studied as follows: after one CR degradation reaction, the catalyst was separated and used for the subsequent CR degradation experiment without any treatment. Finally, the D% of each degradation experiment was calculated. 

### 2.5. Kinetics of Dye Degradation

In order to better analyze the kinetics for the removal of CR, the pseudo-first-order and pseudo-second-order kinetic models were utilized to analyze the data, using the following Equations (2) and (3):

(2) $$\log\left(Q_{e1}-Q_{t}\right)=logQ_{e1}-\frac{k_{1}t}{2.303}$$

(3) $$\frac{t}{Q_{t}}=\frac{1}{k_{2}Q_{e2}^{2}}+\frac{t}{Q_{e2}}$$

Herein, *Q_e_* (mg/g) and *Q_t_* (mg/g) mean the CR removal amount at equilibrium and time *t* (min); *k*_1_ and *k*_2_ mean the kinetic constant of pseudo-first-order kinetic models and pseudo-second-order kinetic models, respectively.

## 3. Results and Discussion

### 3.1. Characterization of CDM

**Figure 1:**
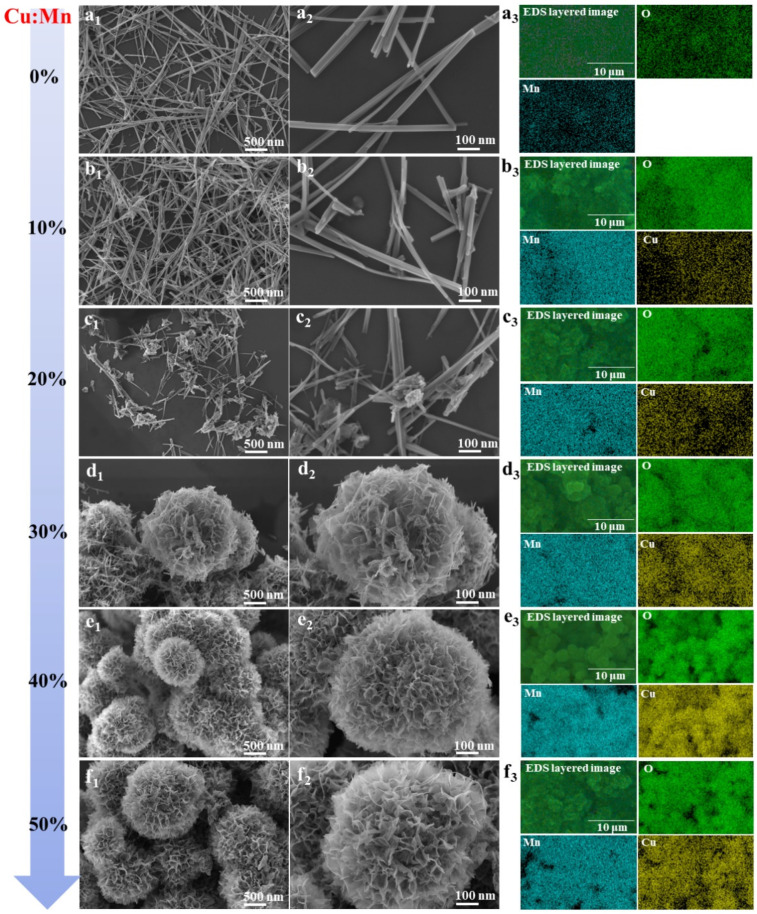
SEM images and EDS elemental maps of CDM catalysts: (**a**) 0%-CDM, (**b**) 10%-CDM, (**c**) 20%-CDM, (**d**) 30%-CDM, (**e**) 40%-CDM, and (**f**) 50%-CDM.

The morphologies of CDM catalysts were investigated and are shown in Figure 1. It can be seen that 0%-CDM contained nanowires with a diameter of about 20 nm, but the structure of the nanowires began to become irregular when Cu was introduced, just as shown in Figure 1b. When the Cu/Mn molar ratio increased to 20%, there were some irregular clumps that appeared in addition to the nanowires, and these clumps were bound to the nanowires. More surprisingly, a new flower-like morphology consisting of shorter nanowires, generated as the Cu/Mn molar ratio, increased to 30%. As the content of Cu increased continually, the flower-like morphology became more regular and the “petal” parts appeared thinner, and there were no obvious nanowires that could be seen in the flower-like nanocatalyst (Figure 1e,f). In addition, the EDS elemental maps proved that O, Mn, and Cu elements were evenly distributed in the CDM and the content of Cu in catalysts increased significantly from 10%-CDM to 50%-CDM, and the concrete content of Cu is given in Table S1. Therefore, the morphologies of nanocatalysts were affected greatly by the copper doping.

**Figure 2:**
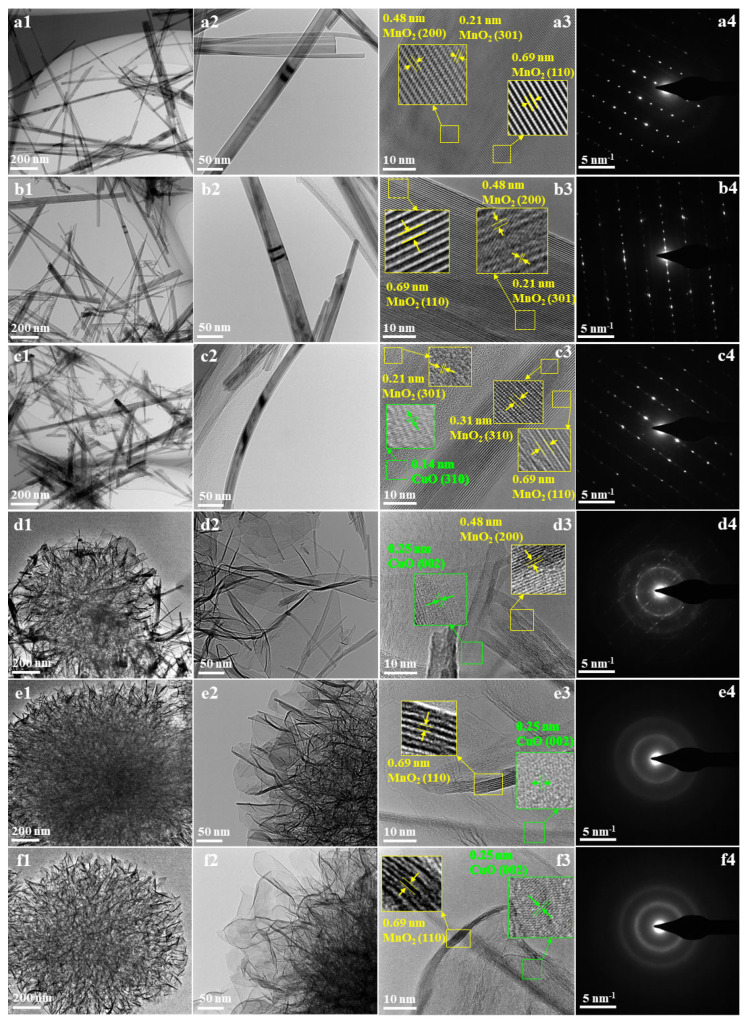
TEM images (**a1**–**f1**, **a2**–**f2**), HRTEM images (**a3**–**f3**) and SAED patterns (**a4**–**f4**) of CDM catalyst: (**a**) 0%-CDM, (**b**) 10%-CDM, (**c**) 20%-CDM, (**d**) 30%-CDM, (**e**) 40%-CDM, (**f**) 50%-CDM.

In order to investigate the morphologies and crystal structures of CDM, TEM and SAED were measured and are given in Figure 2. In terms of morphologies, the results are consistent with the previous SEM characterization results. With increasing Cu content, the morphology of CDM changes from nanowires to flower-like materials, and the greater the content of Cu, the more regular the shape of the flower-like material. In Figure 2(a3,b3), it is seen that the resolved lattice spacings of 0.69 nm, 0.48 nm, and 0.21 nm belong to the crystal plane (110), (200), (301) of α-MnO_2_. In addition to the lattice structure of α-MnO_2_, the crystal plane structure of CuO was also observed in Figure 2(c3–f3) with an increasing content of Cu; for example, a lattice spacing of 0.14 nm corresponded to CuO (310) and a lattice spacing of 0.25 nm corresponded to CuO (002) [25]. Additionally, some diffraction points were detected by SAED characterizations and are shown in Figure 2(a4–c4), confirming that the 0%-CDM, 10%-CDM, and 20%-CDM are single crystal structures. As shown in Figure 2(d4), both diffraction points and diffraction rings were observed, which means a mixed state of single crystals and polycrystals was formed in 30%-CDM. Furthermore, only diffraction rings appeared in Figure 2(e4,f4), and this proved that only polycrystals were generated in 40%-CDM and 50%-CDM. Moreover, the diffraction rings of 40%-CDM and 50%-CDM displayed a little fuzz, which proves that there were also amorphous structures in these two nanocatalysts, aside from polycrystalline structures. Likewise, the similar amorphous structures also could be found in the thin “petal” parts according to the HRTEM images in Figure 2(e3,f3). Based on the above results, the copper was successfully doped into MnO_2_ and coexisted with MnO_2_ as CuO, and the crystal structure of the catalyst changed from a single crystal to the coexistence of polycrystalline and amorphous structures with an increasing content of copper.

**Figure 3:**
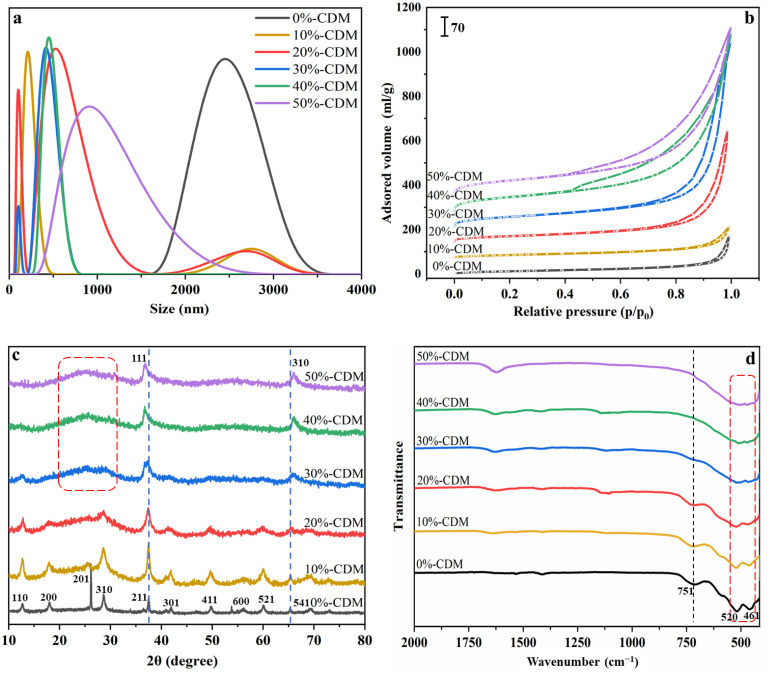
(**a**) Size distribution curve, (**b**) N_2_ adsorption–desorption isotherms, (**c**) XRD patterns, and (**d**) FT-IR spectra of as-prepared CDM.

A laser particle size analyzer was used to characterize the size of CDM and the results are given in Figure 3a. Among them, three samples, containing 0%-CDM, 40%-CDM, and 50%-CDM, showed unimodal particle size distribution, while the other three samples (10%-CDM, 20%-CDM, and 30%-CDM) showed bimodal particle size distribution. These results were consistent with the morphological characterization from SEM and TEM, with the content of doped copper being increased. Some irregular mixed nanomaterials were formed during the transformation of CDM from nanowires to nanoflowers. For the regular nanowires (0%-CDM) and regular nanoflowers (40%-CDM and 50%-CDM), the particle size distributions were relatively uniform.

The specific surface area and pore size distributions of series CDM were investigated and are shown in Figure 3b and Figure S1, respectively. According to Figure 3b, all six kinds of catalysts displayed typical type IV isotherms with an evident H3-type hysteresis loop, which is a typical feature of mesoporous materials [20,26]. Moreover, type I adsorption isotherms could be found in the isotherms of 20%-CDM, 30%-CDM, 40%-CDM, and 50%-CDM, for which a steep increase in the isotherm at a low pressure range (P/P_0_ = 10^−7^–0.01) was detected, so some micropores were proved to exist in these catalysts [27]. Remarkably, hysteresis loops of the catalysts became larger with increasing contents of doped Cu, demonstrating increased mesopore porosity. Combined with the S_BET_ shown in Table 2, 50%-CDM really displayed the highest specific surface area, which was as high as 259.89 m^2^/g, while that of 0%-CDM was only 45.85 m^2^/g. In addition, the total pore volume of the catalyst became larger after the Cu doping; therefore, more available reactive region space and more effective substrate transportation could be provided, contributing to enhanced catalytic activity [22,28]. Based on the data shown in Table 3 and Figure S1, the most probable particle size Dp* also changed obviously with the doping of Cu; 20%-CDM and 30%-CDM displayed large Dp* (18.00 and 22.05 nm), while the other catalysts displayed smaller Dp* values. The change in pore size is due to the change in crystalline grain stacking structure during the formation of nanocatalysts.

Figure 3c showed the XRD patterns of series CDM. For the pure α-MnO_2_ (0%-CDM), the typical diffraction peaks of crystalline α-MnO_2_ could be found at 2θ = 12.8, 18.1, 26.2, 28.7, 37.6, 41.8, 49.8, 56.4, 60.4, and 69.5, which corresponded to the (110), (200), (201), (310), (211), (301), (411), (600), (521), and (541) planes (PDF#00-044-0141) [29,30]. After being Cu doped, the diffraction peaks of crystalline α-MnO_2_ became weaker, and part of the diffraction peaks of α-MnO_2_ even disappeared when the content of Cu was high enough, such as in 40%-CDM and 50%-CDM. Meanwhile, the characteristic diffraction peaks of CuO appeared obviously at 36.7 and 66.4, corresponding to the (111) and (310) planes (PDF#00-048-1548) [25,31]. In addition to the above sharp crystal diffraction peaks, an amorphous structure also appeared with an increasing content of copper, especially for 50%-CDM, 40%-CDM, and 30%-CDM. Coincidentally, the same results were also obtained in the SAED patterns before. 

The chemical structures of catalysts were tested by FT-IR and are given in Figure 3d. Herein, three characteristic absorption peaks could be found at 715 cm^−1^, 520 cm^−1^, and 461 cm^−1^, which corresponded to the stretching vibrations of Mn-O [18]. With an increasing content of Cu, the peak at 715 cm^−1^ disappeared and a new broad peak appeared in the range of 430–550 cm^−1^, especially in the spectra of 40%-CDM and 50%-CDM, which could be due to the Cu-O bonds formed in the catalysts [32,33]. In addition, the peak at 1625 cm^−1^ was attributed to the -OH group combined on the surface of catalysts.

**Figure 4:**
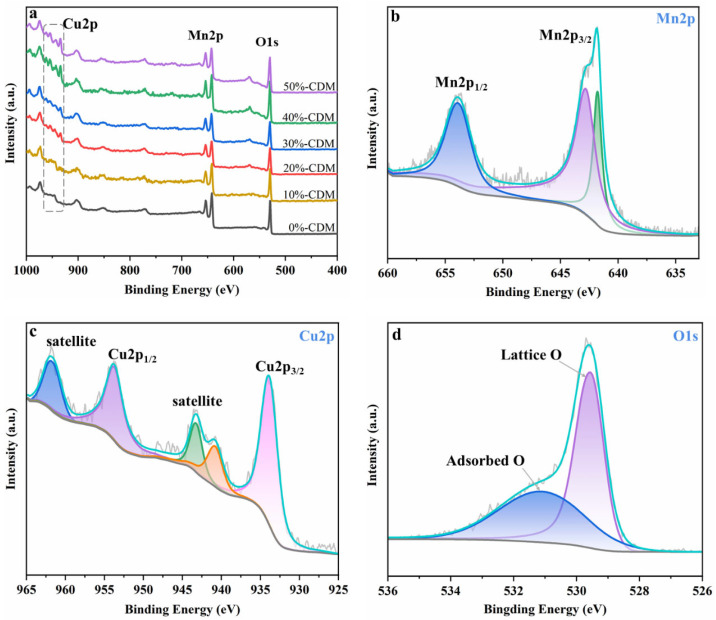
XPS spectra of CDM: (**a**) six CDM; (**b**) Mn 2p on 50%-CDM; (**c**) Cu 2p on 50%-CDM; (**d**) O 1s on 50%-CDM.

XPS measurement was used to study the surface constituents of CDM. According to the XPS broad survey spectra shown in Figure 4a, Mn and O could be found in all of the six CDM, while the characteristic peak of Cu, at the range of 933.9 eV to 962.8 eV, appeared gradually with the increasing Cu/Mn molar ratio; this is to say, it could be found in the survey scan of 20%-CDM, 30%-CDM, 40%-CDM, and 50%-CDM, but not in 0%-CDM and 10%-CDM. Figure 4b shows the spectrum of Mn 2p in 50%-CDM. The peaks appearing at 653.0 eV and 641.3 eV correspond to Mn 2p_1/2_ and Mn 2p_3/2_ [34,35], and the spin separation energy with 11.7 eV indicates the existence of the MnO_2_ phase in 50%-CDM. The Cu 2p spectrum of 50%-CDM is given in Figure 4c, and two obvious peaks appeared at 953.8 eV and 933.9 eV, corresponding to Cu 2p_1/2_ and Cu 2p_3/2_ [36,37], respectively. Additionally, two associated satellite peaks were found at 962.0 eV and 943.2 eV, which proved the presence of Cu^2+^ [25,38]. The O 1s spectrum of 50%-CDM is shown in Figure 4d. Here, two characteristic peaks could be found at 529.6 eV and 531.2 eV, corresponding to lattice O and adsorbed O [39], respectively. Furthermore, the HR-XPS spectra of O 1s for series CDM are illustrated in Figure S2, and the contents of lattice O and adsorbed O in different CDM are given in Table S2. It could be found that the contents of adsorbed O increased obviously with an increasing content of Cu, which signified that greater oxygen vacancy was formed [40], and that the content of oxygen vacancy would affect the catalytic activity of CDM significantly [20]. In particular, the content of adsorbed O in 50%-CDM was a little lower than that in 40%-CDM, meaning that the oxygen vacancy would not be increased if the content of Cu increased continually.

### 3.2. Catalytic Performance of CDM

**Figure 5:**
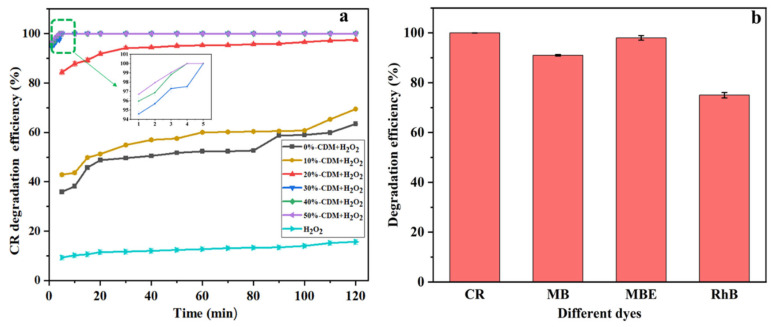
(**a**) Degradation of CR by different CDM catalysts (C_o_ = 60 mg/L, pH = 7, 2 mL 30% H_2_O_2_); (**b**) catalytic performance of M-5 for different dyes (C_o_ = 60 mg/L, pH = 7, 5 min, 2 mL 30% H_2_O_2_).

The series of CDM were used to catalytically degrade CR, and the results are shown in Figure 5a. Firstly, it could be found that the degradation efficiency was as low as 15.7% when there was only H_2_O_2_ added without any catalyst. When CDM and H_2_O_2_ were added simultaneously, it was obvious that 0%-CDM displayed the lowest degradation efficiency, which only reached 63.4% after 120 min. Meanwhile, the degradation efficiency reached 100% after 5 min when 30%-CDM, 40%-CDM, and 50%-CDM were used, and the 50%-CDM displayed the fastest catalytic rate according to the partially enlarged illustration in Figure 5a. Therefore, the catalytic activity of CDM was significantly improved with an increasing amount of Cu doped. This could be explained by the following reasons: firstly, the 50%-CDM displayed the largest specific surface area, which could supply the maximum adsorption and reaction sites for CR; secondly, a greater oxygen vacancy was formed in 50%-CDM according to the XPS results of O1s, although it was lower than that in 40%-CDM. As a result, the catalytic activity of CDM was affected by the specific surface area and oxygen vacancy, simultaneously. Thus, 50%-CDM was selected as the best catalyst and used for the subsequent experiments. 

To evaluate the catalytic performance of 50%-CDM further, degradation experiments of different dyes (CR, MB, MBE, and RhB) were carried out, as displayed in Figure 5b. After 5 min of the reaction, the D% values of CR and MBE reached 100% and 98%, respectively, while the D% values of MB and RhB appeared to be relatively lower and only reached 90% and 75%. The reason may be that CR and MBE are anionic dyes while MB and RhB are cationic dyes, and the 50%-CDM has a positive electrical surface as a metal chemical compound. Therefore, there is a good electrostatic adsorption between anionic dyes (CR, MBE) and 50%-CDM, but there is no such electrostatic adsorption between cationic dyes (MB, RhB) and 50%-CDM. Thus, the 50%-CDM displayed better catalytic activity for CR and MBE. In the subsequent experiment, CR was selected as a target degradation substance.

### 3.3. Optimum Catalytic Conditions of 50%-CDM

**Figure 6:**
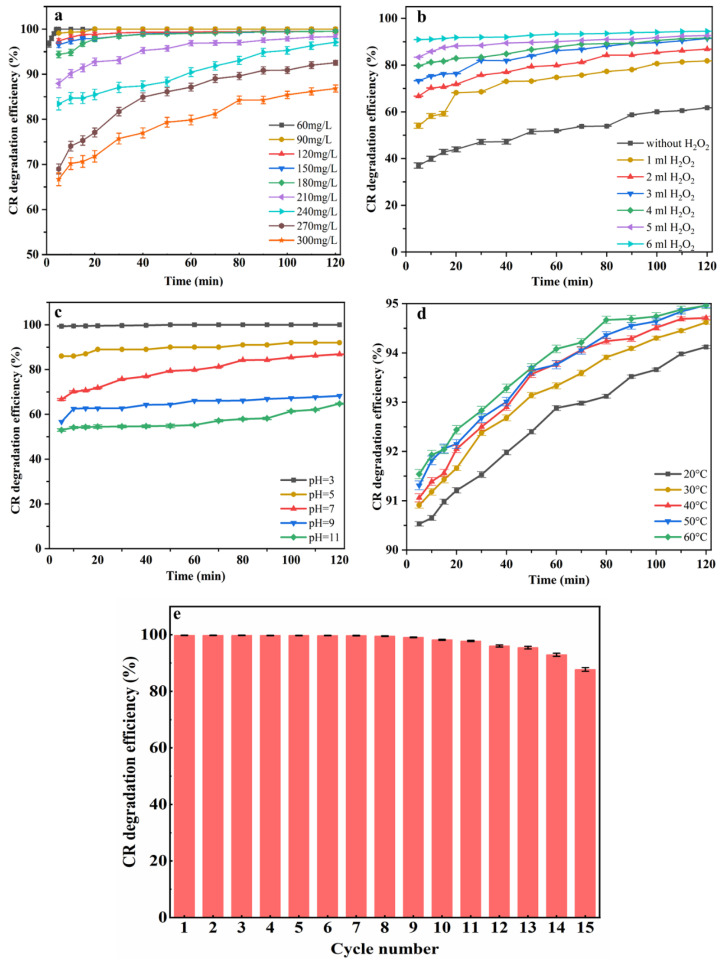
(**a**) Effect of CR concentrations (pH = 7, 2 mL 30% H_2_O_2_); (**b**) effect of dosage of H_2_O_2_ (C_o_ = 300 mg/L, pH = 7); (**c**) effect of pH (C_o_ = 300 mg/L, 2 mL 30% H_2_O_2_); (**d**) effect of temperature (C_o_ = 300 mg/L, pH = 7, 6 mL 30% H_2_O_2_); and (**e**) the reusability of 50%-CDM (C_o_ = 300 mg/L, pH = 3, 6 mL 30% H_2_O_2_, 5 min).

To study the optimum catalytic conditions of CDM, the effects of CR concentration, H_2_O_2_ dosage, pH value, and temperature were investigated, as shown in Figure 6a–d. For a CR solution with a low concentration (60 mg/L), the D% could reach 100% only in 3 min. While the CR concentration increased above 210 mg/L, the D% could not reach 100% in 120 min. Thus, both the CR degradation rate and degradation efficiency decreased when the initial concentration of CR increased. Figure 6b shows the effect of H_2_O_2_ dosage, where it can be seen that the D% of 300 mg/L CR solution only reached 61.7% after 120 min without H_2_O_2_, while it increased to 94.5% when 6 mL H_2_O_2_ was added. In addition, both the degradation rate and degradation efficiency increased obviously with an increasing H_2_O_2_ dosage. When there is no H_2_O_2_, the removal process of CR is only based on physical adsorption. As H_2_O_2_ is added, in addition to physical adsorption, the degradation of CR by a Fenton-like reaction also plays an important role. The improvement in H_2_O_2_ dosage could bring out more active free radicals, which causes a quicker degradation rate and a higher degradation efficiency [23,36]. The pH value always has an important effect on the activity of catalysts, and the influences of the pH value from 3 to 11 on the degradation of CR were studied and are shown in Figure 6c. Herein, the highest catalytic capacity of 50%-CDM was obtained when the pH was 3.0, for which the D% of CR was tested as 99.4% after 5 min, and this is a fairly fast catalytic rate for highly concentrated CR solution. However, the degradation efficiency decreased significantly with an increasing pH value, and the D% decreased as low as 64.7%. The reason for this has been explained in many previous studies, as more free radicals from H_2_O_2_ in a Fenton-like system are generated in acidic conditions [1,36]. The effect of temperature on the degradation efficiency of CR was also investigated. As shown in Figure 6d, the degradation efficiency increased with elevated temperature. However, the differences in D% at different temperatures were not too large, and the difference between the maximum D% and the minimum D% was only 0.4% after 120 min. Thus, the temperature had no significant effect on the catalytic activity of the catalysts.

To evaluate the reusability of 50%-CDM, fifteen consecutive catalytic degradations of CR were carried out. After each run, the catalyst was separated from the solution by centrifugation, and then it was used directly for the next round of catalytic degradation without any more treatments. As shown in Figure 6e, all of the D% values were higher than 99% in the first nine catalytic degradation experiments, while the D% began to decrease from the 10th reuse and the D% dropped to 87% in the 15th reuse. However, all of the D% values were higher than 90% in the first 14 cycles. As a result, 50%-CDM displayed excellent reusability. 

In order to estimate the stability of 50%-CDM, the leaching amount of Cu and Mn in wastewater after the reusability reaction with the 14th reuse was tested by ICP-OES, and the contents of Cu and Mn were detected as 0.0023 mg/L and 0.094 mg/L, which were lower than the concentrations specified in the national standard GB5749-2022 [41] in China (C_Cu_ < 1 mg/L, C_Mn_ < 0.1 mg/L). In addition, SEM and XPS were also used to evaluate the stability of 50%-CDM and the results are given in Figures S3 and S4. It was found that the morphology of the 50%-CDM still maintained a flower-like morphology, but some of the microspheres’ structures collapsed and were no longer regular flower-shaped structures. According to the XPS broad survey spectra, Mn, O, and Cu are all found in Figure S4a. The characteristic peaks of Cu and Mn almost remained consistent with those of the original 50%-CDM in Figure 4b,c. Notably, the O 1s spectrum changed obviously and two new characteristic peaks appeared at 531.9 eV and 533.6 eV, which correspond to the organic C-O and organic C=O, and these may be from the degradation products of CR. Based on the above results, the 50%-CDM displayed good stability during the application.

**Figure 7:**
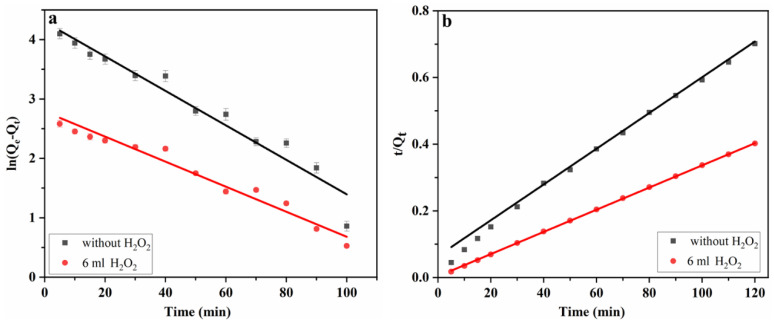
(**a**) Pseudo-first-order kinetics model and (**b**) pseudo-second-order kinetics model with different H_2_O_2_ dosages.

To better analyze the kinetics for the removal of CR, two kinetic models including a pseudo-first-order model and pseudo-second-order model were utilized to fit the experimental data with H_2_O_2_ and without H_2_O_2_, as shown in Figure 7a,b, and the kinetic model rate constants are listed in Table 3. Obviously, the R^2^ of the pseudo-first-order was relatively low (0.9448, 0.9608), whether with or without H_2_O_2_, while that of the pseudo-second-order model (0.9958, 0.9999) was higher. Thus, the removal process of CR was more consistent with the pseudo-second-order kinetics model. Additionally, the Qe calculated in the pseudo-second-order model was closer to the Qe obtained in experiments (171.15 mg·g^−1^ without H_2_O_2_ and 298.46 mg·g^−1^ with 6 mL H_2_O_2_). 

In Table 4, the catalytic performances of other catalysts reported in previous studies are compared with that of 50%-CDM. Obviously, the catalyst prepared here displayed an excellent degradation efficiency, for either low-concentration CR solution or high-concentration CR solution. It is noteworthy that such an impressive catalytic performance has been rarely reported in previous studies. 

### 3.4. Mechanism

**Figure 8:**
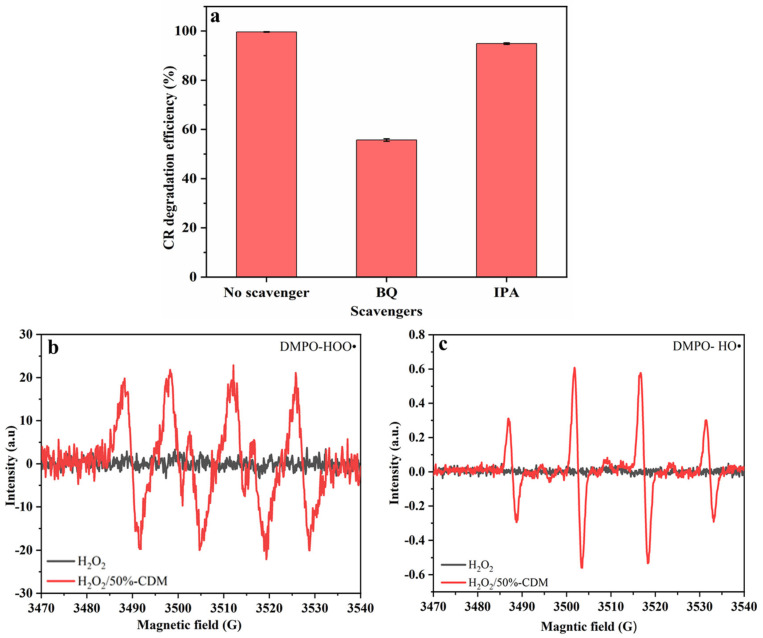
(**a**) The degradation efficiency of CR in the presence of scavengers (C_o_ = 300 mg/L, pH = 3, 6 mL 30% H_2_O_2_, 5 min), (**b**) EPR signals of DMPO-HO• adducts, and (**c**) EPR signals of DMPO-HOO• adducts.

In order to investigate the catalytic mechanism of CDM, free radical scavenging experiments were carried out by using p-benzoquinone (BQ) and isopropyl alcohol (IPA) as the scavengers for HOO• and HO•, respectively. As shown in Figure 8a, the D% decreased from 99.6% to 55.7% when BQ was added, while the D% only decreased to 94.9% when IPA was added. As a result, both HOO• and HO• played a role during the degradation process, but HOO• played a greater role apparently. 

Furthermore, the EPR measurement was utilized to detect the existence of HOO• and HO• in the Fenton-like reaction. As shown in Figure 8b,c, both DMPO-HOO• (1:1:1:1) signal peaks [51] and DMPO-HO• (1:2:2:1) signal peaks [52,53] were obtained with the existence of 50%-CDM and H_2_O_2_, but no peaks appeared as only H_2_O_2_ exist but there was an absence of H_2_O_2_. However, the signal intensity of HO• was significantly lower than that of HOO• under the same test conditions. Thus, HOO• played a greater role in the Fenton-like reaction, which was consistent with the results obtained in the above free radical scavenging experiments.

**Figure 9:**
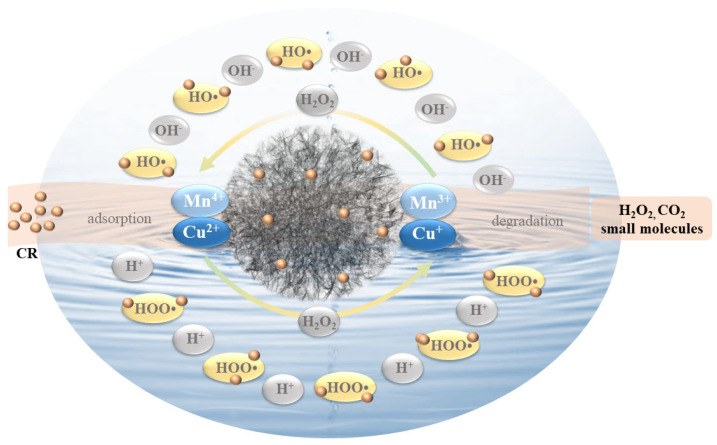
The probable degradation mechanism of CR in the 50%-CDM/H_2_O_2_ catalytic system.

According to the above results, the probable degradation mechanism of CR in the 50%-CDM/H_2_O_2_ catalytic system is illustrated in Figure 9. Based on the large specific surface area of the 50%-CDM (259.89 m^2^/g), CR could be adsorbed onto a flower-like catalyst at first. As shown in Equations (4) and (5), H_2_O_2_ was decomposed to HO• and HOO• along with the valence change in manganese ions and copper ions; thus, CR was oxidized and degraded by the free radical and some non-toxic or low-toxic products such as CO_2_, and H_2_O and other small molecules were produced. Herein, the two metal ions could catalyze H_2_O_2_ to generate free radicals in the 50%-CDM/H_2_O_2_ catalytic system, which is also one of the reasons as to why the 50%-CDM displayed the highest catalytic activity. Additionally, copper ions and manganese ions can also promote a mutual valence transition after low-covalent ions (Mn^3+^ and Cu^+^) are generated, as shown in Equations (6) and (7), for which the content of HO• detected is lower that of HOO•, and this theoretical inference is consistent with previous results obtained from EPR and radical scavenger experiments. Finally, the valence changes in metal ions through different methods ensured the regeneration of 50%-CDM, and led to the continual degradation of CR.

(4) $$M_{n}^{4+}/{\mathrm{Cu}}^{2+}+H_{2}O_{2}\to M_{n}^{3+}/{\mathrm{Cu}}^{+}+\mathrm{HOO}\cdot +H^{+}$$

(5) $$M_{n}^{3+}/{\mathrm{Cu}}^{+}+H_{2}O_{2}\to M_{n}^{4+}/{\mathrm{Cu}}^{2+}+\mathrm{HO}\cdot +{\mathrm{OH}}^{-}$$

(6) $$M_{n}^{3+}+{\mathrm{Cu}}^{2+}\to M_{n}^{4+}+{\mathrm{Cu}}^{+}$$

(7) $$M_{n}^{4+}+{\mathrm{Cu}}^{+}\to M_{n}^{3+}+{\mathrm{Cu}}^{2+}$$

## 4. Conclusions

In this study, Cu-doped manganese dioxide nanocatalysts with different Cu/Mn molar ratios were prepared with a simple hydrothermal reaction. Evidently, the Cu/Mn molar ratio had a significant effect on the structure and catalytic performance of CDM, whereas 50%-CDM, a flower-like nanocatalyst, with the highest content of Cu doped displayed the best catalytic activity due to the largest specific surface area and abundant oxygen vacancy. When 50%-CDM was used to degrade CR wastewater, an impressive degradation efficiency was obtained; for example, the D% reached 100% for 60 mg/L CR in 3 min at neutral conditions and the D% also reached 99.4% for 300 mg/L CR in 5 min at acidic conditions. Additionally, 50%-CDM also displayed excellent reusability, and the D% remained higher than 99% in nine cycles and higher than 90% in fourteen cycles. Finally, combing the high specific surface area and oxygen vacancy of the flower-like catalyst, a highly reactive and reusable heterogeneous Fenton catalyst was obtained in this work, which could be promising for practical dye wastewater treatment.

## Figures and Tables

**Table 1:** Series of nanocatalysts.

Samples	0%-CDM	10%-CDM	20%-CDM	30%-CDM	40%-CDM	50%-CDM
Cu:Mn	1:0	1:10	1:5	1:3.3	1:2.5	1:2

Note: Cu:Mn means the Cu/Mn molar ratio.

**Table 2:** Texture parameters from BET and BJH method.

Samples	S_BET_ (m^2^/g)	Vp (cm^3^/g)	Dp (nm)	Dp* (nm)
0%-CDM	45.85	0.26	16.50	2.04
10%-CDM	57.12	0.22	11.97	2.04
20%-CDM	107.83	0.78	20.90	18.00
30%-CDM	176.89	1.28	20.05	22.05
40%-CDM	243.79	0.93	8.90	3.62
50%-CDM	259.89	1.18	11.37	3.55

Note: S_BET_ means the specific surface area calculated by BET multi-point method; Vp, Dp, and Dp* were calculated by the BJH method, Vp means the total pore volume, Dp means the mean diameter, and Dp* means the most probable particle size, which corresponds to the peak position of the pore size distribution curve.

**Table 3:** Kinetic parameters of CR degradation.

Model	Constant	0 mL H_2_O_2_	6 mL H_2_O_2_
Pseudo-first-order model	R^2^	0.9448	0.9608
	Q_e cal_ (mg g^−1^)	86.90	146.28
	K_1_ (min^−1^)	0.0290	0.0210
Pseudo-second-order model	R^2^	0.9958	0.9999
	Q_e cal_ (mg g^−1^)	175.44	294.12
	K_2_ (min^−1^)	0.0054	0.0033

Note: Q_e cal_ means the maximum removal amount calculated by kinetic models.

**Table 4:** Comparison of the performance of catalysts in previous studies.

Catalyst.	Dye	C_o_ (mg/L)	Degradation Method	Time (min)	D_dye_ (%)	Ref.
Biochar-CuO	MB	10	Periodate-AOP	30	100	[42] 2023
CuO	RhB	50	PMS-AOP	60	100	[43] 2023
Fe_3_O_4_/Biochar	CR	100	PMS-AOP	60	94.3	[44] 2023
Fe_3_O_4_/biochar	AO 7	200	photo-Fenton	120	100	[45] 2023
RuSA-N-C	AO 7	90	Fenton-like	30	100	[46] 2023
3D-MnO_2_	AO 7	50	PMS-AOP	5	98.3	[16] 2022
ZnS	CR	50	photocatalytic	120	94.8	[47] 2022
ZnO/ZnS@AWA	CR	40	photocatalytic	150	98.8	[48] 2022
SnO_2_-CdS	CR	10	photocatalytic	40	83.0	[49] 2022
MnO_2_-ceramsite	AO 7	100	Fenton-like	60	85.2	[50] 2021
Cu doped MnO_2_	CR	60	Fenton-like	3	100	This work
		300		5	99.4	

Note: AO 7 means acid orange 7.

## Data Availability

Data are contained within the article and Supplementary Materials.

## Supplementary Materials

The following supporting information can be downloaded at: https://www.mdpi.com/article/10.3390/nano14100833/s1, Table S1. Elemental content of catalyst obtained from EDS; Figure S1. Pore size distributions of series CDM: (**a**) 0%-CDM; (**b**) 10%-CDM; (**c**) 20%-CDM; (**d**) 30%-CDM; (**e**) 40%-CDM; (**f**) 50%-CDM; Figure S2. HR-XPS spectra of O 1s for series CDM; Table S2. The atomic% of lattice O and adsorbed O obtained from XPS; Figure S3. SEM images and EDS elemental maps of 50%-CDM after reused 14th; Figure S4. XPS spectra of 50%-CDM after reused 14th.
