# Supplementary material for: Preparation of Heterogeneous Fenton Catalysts Cu-Doped MnO2 for Enhanced Degradation of Dyes in Wastewater
Source: Nanomaterials (Basel). 2024 May 9;14(10):833. doi: 10.3390/nano14100833 (PMC11124159; doi:10.3390/nano14100833)
Supplement: Supplementary file 1 [file nanomaterials-14-00833-s001.zip › nanomaterials-2968313-supplementary.pdf]

Preparation of Heterogeneous Fenton Catalysts Cu-Doped MnO_2 for Enhanced Degradation of Dyes in Wastewater

Xiao Liu [1,2,3,*], Lu Wang [1], Jiran Li [1], Rong Li [1], Runze He [1], Wanglong Gao [1] and Neng Yu [4]

[1] School of Materials Science & Engineering, North Minzu University, Yinchuan 750021, China
[2] Key Laboratory of Polymer Materials and Manufacturing Technology, North Minzu University, Yinchuan 750021, China
[3] International Scientific and Technological Cooperation Base of Industrial Waste Recycling and Advanced Materials, Yinchuan 750021, China
[4] Huadian Electric Power Research Institute Company, Hangzhou 310012, China
* Correspondence: plzxlx@163.com

Table S1 Elemental content of catalyst obtained from EDS

element	0%-CDM	10%-CDM	20%-CDM	30%-CDM	40%-CDM	50%-CDM
O	34.67	30.23	28.88	29.59	29.06	28.42
Mn	65.33	63.27	59.19	54.78	50.13	50.66
Cu	0	6.50	11.93	15.63	20.81	20.92

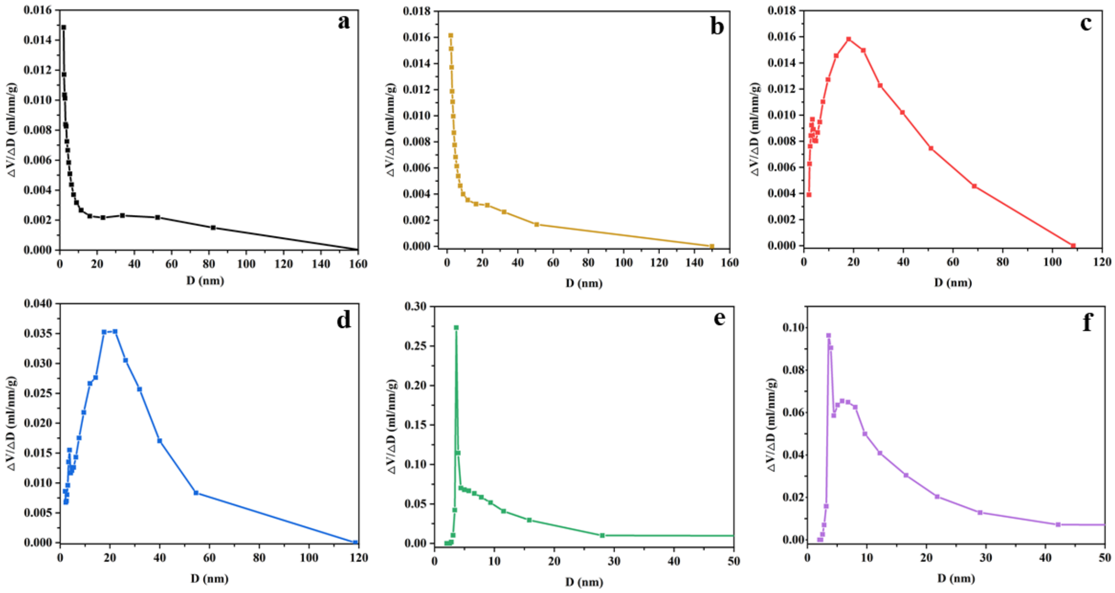

Figure S1. Pore size distributions of series CDM: (a) 0%-CDM; (b) 10%-CDM; (d) 20%-CDM; (d) 30%-CDM; (e) 40%-CDM; (f) 50%-CDM;

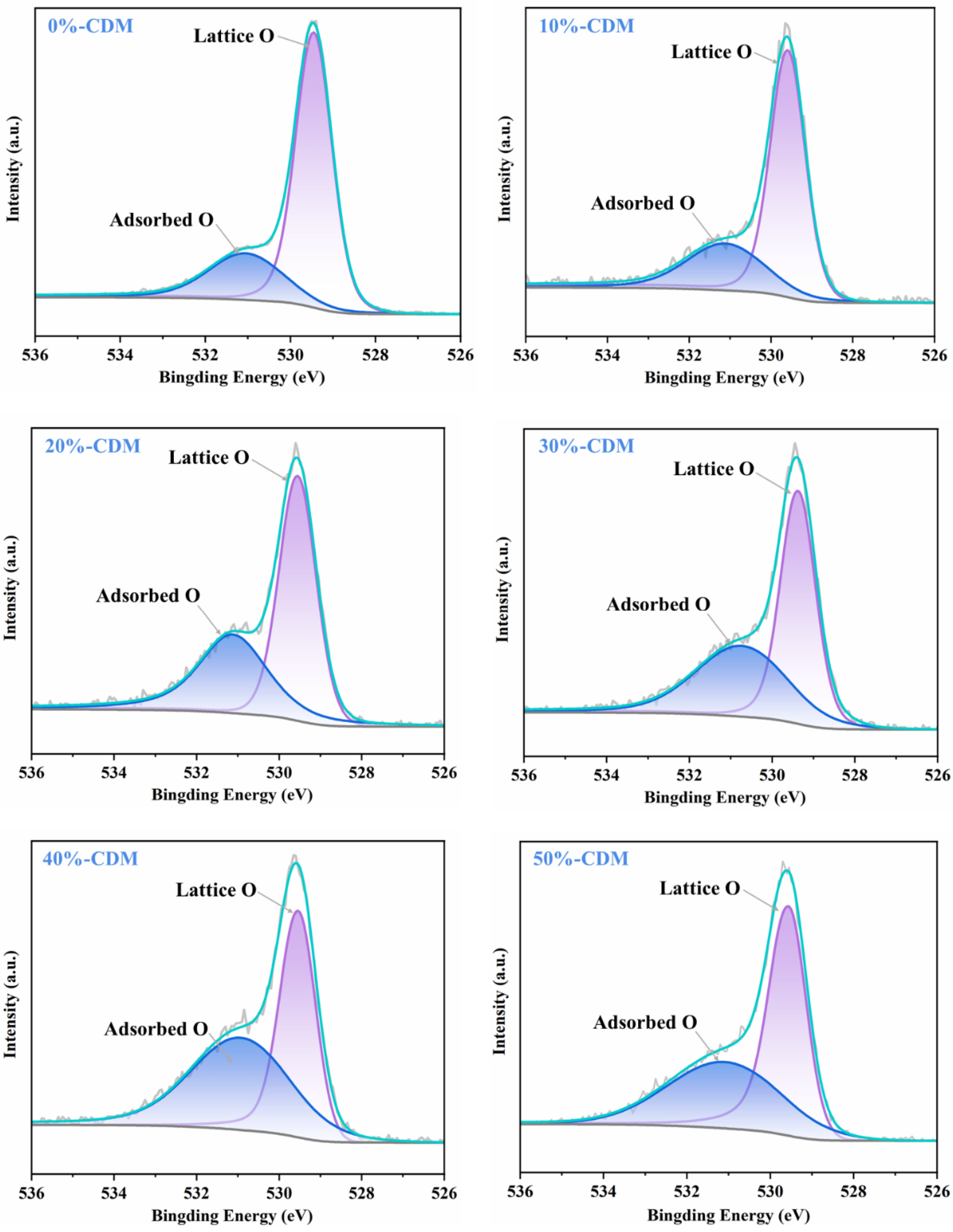

Figure S2. HR-XPS spectra of O 1s for series CDM.

Table S2. the atomic% of lattice O and adsorbed O obtained from XPS

	0%-CDM	10%-CDM	20%-CDM	30%-CDM	40%-CDM	50%-CDM
Lattice O	74.69%	71.87%	60.77%	56.64%	49.55%	51.73%
Adsorbed O	25.31%	28.13%	39.23%	43.36%	50.45%	48.27%

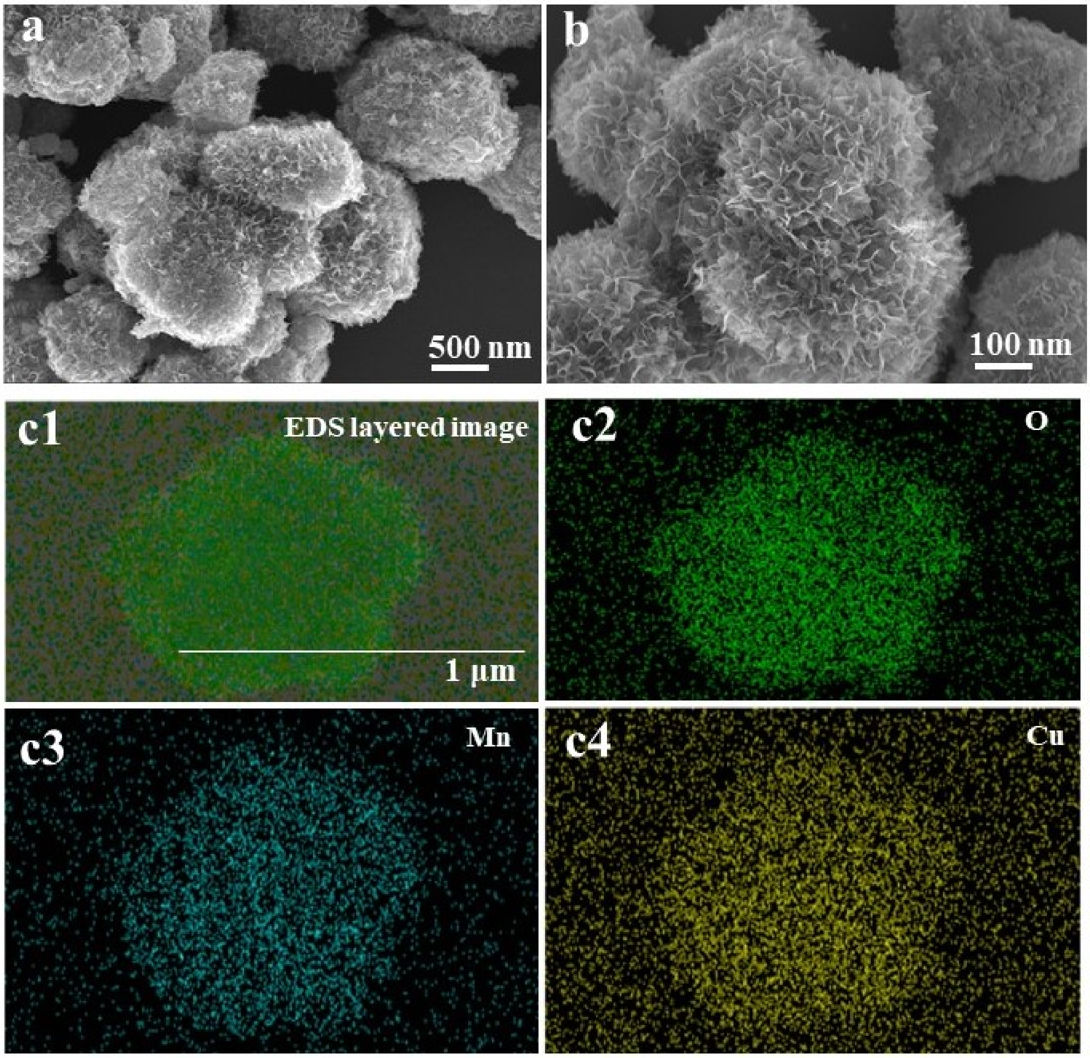

Figure S3. SEM images and EDS elemental maps of 50%-CDM after reused 14th.

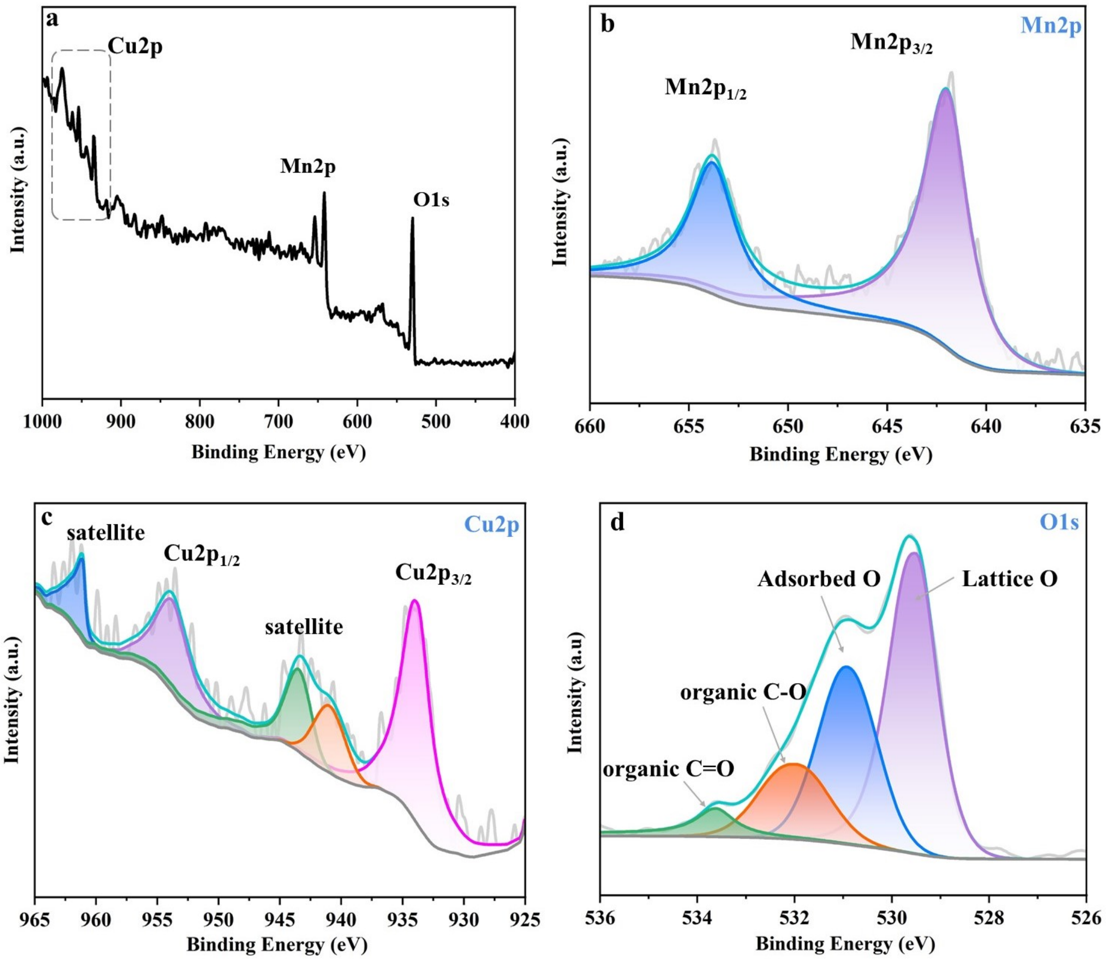

Figure S4. XPS spectra of 50%-CDM after reused 14th.
